# Targeting Endothelial Dysfunction in Eight Extreme-Critically Ill Patients with COVID-19 Using the Anti-Adrenomedullin Antibody Adrecizumab (HAM8101)

**DOI:** 10.3390/biom10081171

**Published:** 2020-08-11

**Authors:** Mahir Karakas, Dominik Jarczak, Martin Becker, Kevin Roedl, Marylyn M. Addo, Frauke Hein, Andreas Bergmann, Jens Zimmermann, Tim-Philipp Simon, Gernot Marx, Marc Lütgehetmann, Axel Nierhaus, Stefan Kluge

**Affiliations:** 1 German Center for Cardiovascular Research (DZHK), Partner Site Hamburg-Kiel-Lübeck, 20251 Hamburg, Germany; 2 Department of Cardiology, University Heart & Vascular Center Hamburg, 20251 Hamburg, Germany; martin.becker@stud.uke.uni-hamburg.de; 3 Department of Intensive Care Medicine, University Medical Center Hamburg-Eppendorf, 20251 Hamburg, Germany; d.jarczak@uke.de (D.J.); k.roedl@uke.de (K.R.); nierhaus@uke.de (A.N.); s.kluge@uke.de (S.K.); 4 Division of Infectious Diseases, First Department of Medicine, University Medical Center Hamburg-Eppendorf, 20251 Hamburg, Germany; m.addo@uke.de; 5 Department of Clinical Immunology of Infectious Diseases, Bernhard Nocht Institute for Tropical Medicine, 20359 Hamburg, Germany; 6 German Center for Infection Research (DZIF), Partner Site Hamburg-Lübeck-Borstel-Riems, 20359 Hamburg, Germany; 7 Adrenomed AG, 16761 Hennigsdorf, Germany; fhein@adrenomed.com (F.H.); bergmann@sphingotec.de (A.B.); jzimmermann@adrenomed.com (J.Z.); 8 SphingoTec GmbH, 16761 Hennigsdorf, Germany; 9 4TEEN4 Pharmaceuticals GmbH, 16761 Hennigsdorf, Germany; 10 Department of Intensive Care and Intermediate Care, University Hospital RWTH Aachen, 52074 Aachen, Germany; tsimon@ukaachen.de (T.-P.S.); gmarx@ukaachen.de (G.M.); 11 Institute of Medical Microbiology, Virology and Hygiene, University Medical Center Hamburg-Eppendorf, 20251 Hamburg, Germany; mluetgehetmann@uke.edu

**Keywords:** COVID-19, Adrecizumab, HAM 8101, adrenomedullin, endothelial function

## Abstract

Recently, the stabilization of the endothelium has been explicitly identified as a therapeutic goal in coronavirus disease 2019 (COVID-19). Adrecizumab (HAM8101) is a first-in-class humanized monoclonal anti-Adrenomedullin (anti-ADM) antibody, targeting the sepsis- and inflammation-based vascular and capillary leakage. Within a “treatment on a named-patient basis” approach, Adrecizumab was administered to eight extreme-critically ill COVID-19 patients with acute respiratory distress syndrome (ARDS). The patients received a single dose of Adrecizumab, which was administered between 1 and 3 days after the initiation of mechanical ventilation. The SOFA (median 12.5) and SAPS-II (median 39) scores clearly documented the population at highest risk. Moreover, six of the patients suffered from acute renal failure, of whom five needed renal replacement therapy. The length of follow-up ranged between 13 and 27 days. Following the Adrecizumab administration, one patient in the low-dose group died at day 4 due to fulminant pulmonary embolism, while four were in stable condition, and three were discharged from the intensive care unit (ICU). Within 12 days, the SOFA score, as well as the disease severity score (range 0–16, mirroring critical resources in the ICU, with higher scores indicating more severe illness), decreased in five out of the seven surviving patients (in all high-dose patients). The PaO2/FiO2 increased within 12 days, while the inflammatory parameters C-reactive protein, procalcitonin, and interleukin-6 decreased. Importantly, the mortality was lower than expected and calculated by the SOFA score. In conclusion, in this preliminary uncontrolled case series of eight shock patients with life-threatening COVID-19 and ARDS, the administration of Adrecizumab was followed by a favorable outcome. Although the non-controlled design and the small sample size preclude any definitive statement about the potential efficacy of Adrecizumab in critically ill COVID-19 patients, the results of this case series are encouraging.

## 1. Introduction

Beginning in December 2019, a novel coronavirus, designated SARS-CoV-2, caused an international outbreak of a respiratory illness termed COVID-19 [1,2]. Its full spectrum ranges from a mild, self-limiting respiratory tract illness to severe progressive pneumonia, multi-organ failure, and death [3] Thus far, there are no specific therapeutic agents for coronavirus infections, and a causal therapy beyond supportive measures is not available [4]. The main reason for admission to the ICU is acute hypoxemic respiratory failure, which often requires mechanical ventilation, with a high mortality [5].

Adrenomedullin is described as a key player in the (dys-) regulation of endothelial function and vascular integrity [6]. Adrecizumab is a first-in-class humanized monoclonal anti-ADM antibody that only marginally inhibits ADM activity but enhances its half-life and thus acts as a long-lasting plasma ADM enhancer, stabilizing and maintaining the endothelial barrier function [7]. By increasing the functional plasma ADM levels, Adrecizumab is hypothesized to target the sepsis- and inflammation-based vascular and capillary leakage. The latter leads to the deterioration of severe COVID-19 to septic shock and ARDS; a recent study used electron microscopy examinations in the autopsy lung tissues of COVID-19 patients, and found the diffuse loosening of the inter-endothelial junctional complex [8]. The rationale for the use of Adrecizumab is derived from the biomarker-guided phase-2 sepsis trial AdrenOSS-2, which just announced positive top-line results [9,10]. Moreover, very recently, the stabilization of the endothelium has been explicitly identified as a therapeutic goal in COVID-19 [11]. As derived from the AdrenOSS-1 and the AdrenOSS-2 studies, the administration of Adrecizumab is conducted in a biomarker-guided way, indicated by an elevated “do-treat-if high” biomarker bio-ADM (bioactive ADM) and a low-to-normal “do-not-treat-if-high” biomarker DPP3 [9,10,12,13,14]. Bio-ADM is a biomarker for endothelial function; it shows an inverse relationship with blood pressure and subsequently a direct relationship with vasopressor demand [9]. DPP3 is an amino dipeptidase involved in the degradation process of renin-angiotensin system (RAS) peptides, especially angiotensin II [12]. The DPP3 blood levels in septic and cardiogenic shock patients at admission are associated with severe organ dysfunction, refractory shock, and high short-term mortality [12,13]. Furthermore, a reduction in the DPP3 levels within 24 h of admission was associated with improved outcomes in these patient populations, and, similar to the SARS-CoV-2 receptor ACE2, DPP3 mediates the degradation of Ang II [14,15]. Accordingly, these assumptions strengthen the hypothesis that improving endothelial function by the modification of the ADM homeostasis might improve the prognosis and outcomes in severely affected COVID-19 patients.

So far, several experimental therapies have been evaluated in the critically ill, while data on experimental therapies in extreme-critically ill patients is sparse [1]. Here, we describe the first clinical experience with Adrecizumab in extreme-critically ill patients with COVID-19.

## 2. Materials and Methods

### 2.1. Regulatory

The treatment was conducted at the Department of Intensive Care Medicine, University Medical Center Hamburg-Eppendorf in Hamburg, Germany. The clinical outcomes were compared before and after the application of Adrecizumab. The treatment on a named-patient basis approach is based on article 37 of the Declaration of Helsinki and article 41 of the German Pharmaceuticals Act [16,17]. Accordingly, while ethics committee approval and approval by regulatory bodies are not applicable for this approach, both institutions were notified, and informed consent was obtained from the patients, their relatives, or legal representatives.

### 2.2. Patients

Patients with laboratory-confirmed COVID-19, classified as critically ill, were assessed for Adrecizumab treatment when meeting the following criteria: (i) ARDS, (ii) PaO2/FiO2 ≤ 220 (PaO2 measured in mmHg and FiO2 measured as the fraction of inspired oxygen), (iii) mechanical ventilation, (iv) hypotension with vasopressors required to maintain a mean arterial pressure of ≥65 mmHg, (v) acute clinical deterioration, (vi) a circulating bio-ADM plasma level of ≥60 pg/mL or a ≥25% relative increase within 24 h, and (vii) circulating DPP3 plasma levels of ≤50 ng/mL.

### 2.3. Laboratory Diagnostics

Lactate, C-reactive protein (CRP), procalcitonin, and interleukin-6 were determined in the routine lab. SARS-CoV-2 was detected on a high-throughput platform, the Roche Cobas 6800 (Roche Diagnostics, Basel, Switzerland), using the “open channel” for the integration of a laboratory-developed assay, as recently published [18]. DPP-3 was measured using the IB10 sphingotest^®^ DPP3, a commercial point-of-care immunoassay from SphingoTec GmbH/4TEEN4 Pharmaceuticals (Hennigsdorf, Germany). Bio-ADM was measured in plasma using a commercial immunoassay from SphingoTec GmbH (sphingotest^®^ bio-ADM, Hennigsdorf, Germany) [9,10].

### 2.4. Administration of Adrecizumab

Patients received a single dose of Adrecizumab at a dose of 4 mg/kg (2 patients) or 8 mg/kg (6 patients) body weight over a 1-h period. The dose selection was based on the baseline bio-ADM levels, the clinical course within the last few hours, and acute deterioration. The dose range was between 400 mg and 1000 mg. Adrecizumab was administered between 1 and 3 days after the initiation of mechanical ventilation, separately from any concomitant drugs using a dedicated lumen of a central venous catheter or a separate peripheral line.

### 2.5. Disease Severity Classification

Disease severity was classified using the SOFA and SAPS-II scores. The dedicated disease severity score aims to mirror critical resources on ICU, and therefore considers the following 4 dimensions: (i) vital and hospitalization status, (ii) circulation status, (iii) ventilation status, and (iv) the PaO2/FiO2-index. The maximum score is 16, while the minimum is 0, with higher scores indicating a more severe illness.

### 2.6. Clinical Information

Clinical information for the 8 patients before and after the Adrecizumab administration was obtained from the hospital information system and included the following: demographic data, including anamnesis and physical examination; treatment and therapies, including mechanical ventilation and antiviral therapies; clinical data, including PaO2/FiO2, the SOFA score (range 0–24, with higher scores indicating a more severe illness), and the SAPS-II score (higher scores indicating a more severe illness); and laboratory data, including bio-ADM, DPP3, the white blood cell count, lactate, the liver and kidney function, and inflammatory factors (CRP, procalcitonin, and interleukin-6).

## 3. Results

The clinical characteristics of the patients are summarized in Table 1. A total of eight patients were treated with Adrecizumab—seven male patients and one female patient. The patients’ age range was between 31 and 76 years. All eight patients had pre-existing conditions, with six having type 2 diabetes, and seven suffering from hypertension. All the patients were in critical condition, with ARDS and shock. Six out of eight were suffering from renal failure, with five of them in need of renal replacement therapy, and one out of eight suffering from liver failure.

Table 2 shows the dose of Adrecizumab administered and the course of the therapy-guiding biomarkers bio-ADM and DPP3. All eight patients received a single dose of Adrecizumab at a dose of 4 mg/kg (two patients) or 8 mg/kg (six patients) body weight. Dose selection was based on the baseline bio-ADM levels, the clinical course within the last few hours, and the acute deterioration. The dose range was between 400 mg and 1000 mg. The therapy was well tolerated in all patients, and no immediate adverse reactions were noted. The median bio-ADM at baseline was 77.8 (IQR 63.9:102.6) pg/mL, while the median DPP-3 was 18.7 [(QR 17.9:19.0) ng/mL. Upon the Adrecizumab administration, the bio-ADM levels strongly increased (median 383.0 (IQR 264.5:403.5) pg/mL).

Table 3 shows the course of clinical parameters and scores before and after the administration of Adrecizumab. Upon the administration of Adrecizumab, the PaO2/FiO2 and SOFA score improved within 12 days in five patients.

**Figure 1:**
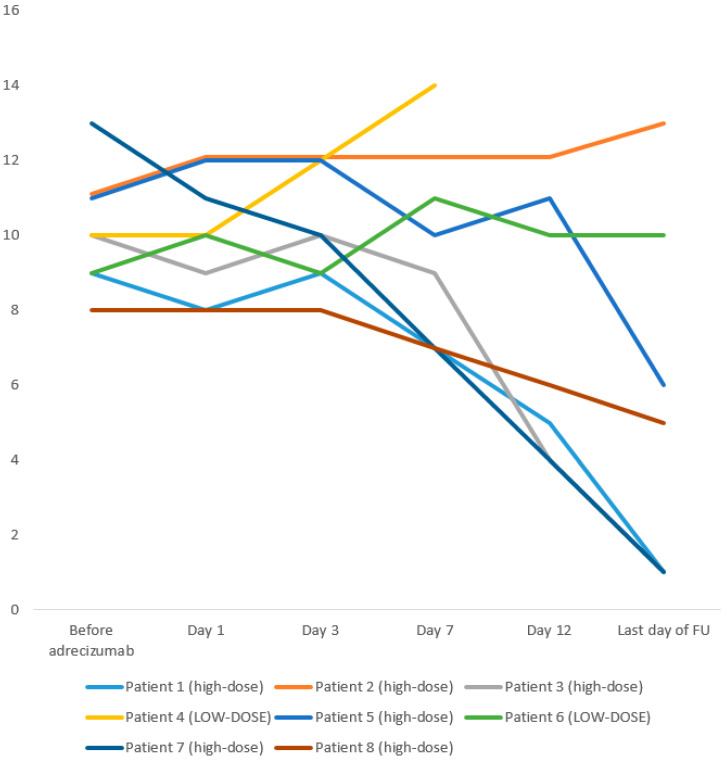
Change in the clinical severity score during follow-up. FU = follow-up; ICU = intensive care unit; VA = veno-arterial; VV =veno-venous. Scoring system: maximum 16, minimum 0-with higher scores indicating more severe illness. Vital and hospitalization status: deceased (5), on ICU ward (3), on normal ward (1), discharged (0). Circulation: VA-ECMO (4), max. noradrenaline > 0.40 µg/kg/min (3), max. noradrenaline 0.40–0.10 µg/kg/min (2), max. noradrenaline < 0.10 µg/kg/min (1), no vasopressor (0). Ventilation status: VV-ECMO (4), mechanical ventilation BIPAP (3), intermittent mechanical ventilation CPAP (2), non-invasive ventilation (1), no ventilation at all (invasive/non-invasive) (0). Mean PaO2/FiO2: ≤100 (3), 101–200 (2), 201–300 (1), >300 (0).

The course of the disease severity score before and after the Adrecizumab administration is shown in Table 4 and Figure 1. Upon therapy, a normalization was seen in three patients, while two others showed a marked decrease. One patient without improvement, as well as the deceased patient, were in the low-dose group. This is an important finding, since this score aims to mirror the critical resources in the ICU.

**Figure 2:**
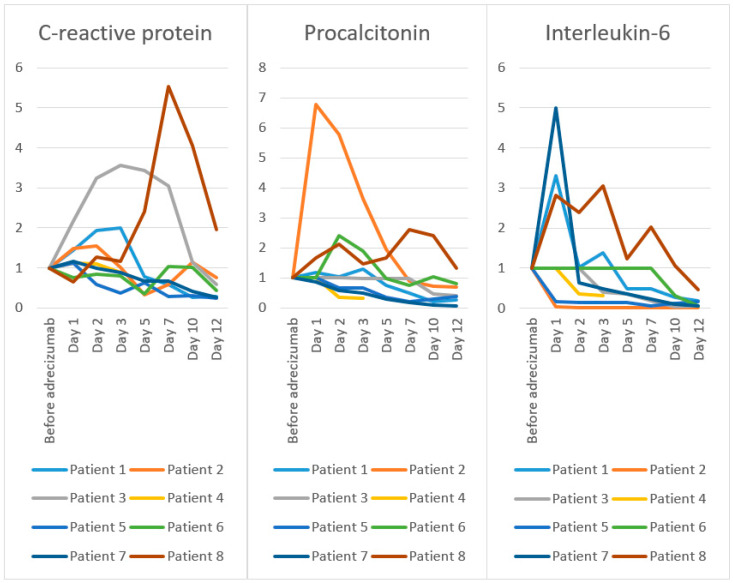
Change in inflammatory parameters (baseline levels normalized to 1) during follow-up.

Figure 2 shows the course of the inflammatory parameters CRP, procalcitonin, and interleukin-6 normalized to the value 1.0 at baseline. While a marked decrease in the interleukin-6 levels was noted in all seven surviving patients, a relevant decrease in the CRP and procalcitonin within 12 days following the Adrecizumab administration was seen in six out of seven patients.

## 4. Discussion

### 4.1. Adrecizumab in COVID-19

Within this case series, eight extreme-critically ill COVID-19 patients were treated with the monoclonal antibody Adrecizumab.

All the patients were mechanically ventilated and needed vasopressors to maintain a mean arterial pressure (MAP) ≥ 65 mmHg. To our knowledge, this is the first case series evaluating an experimental therapy in extreme-critically ill COVID-19 patients. The therapy was well tolerated in all the patients, and no immediate adverse reactions were observed. Upon the Adrecizumab administration, the bio-ADM levels significantly increased, clearly documenting efficacy in all the patients. In summary, the administration of the non-neutralizing anti-ADM antibody Adrecizumab was followed by a favorable outcome. Within a short follow-up period of 13 to 27 days, four patients were in stable condition, and three were transferred to a normal ward. Moreover, an encouraging outcome was also seen with regard to the SOFA and disease severity scores, which decreased in five of the seven surviving patients. In addition, the course of PaO2/FiO2 showed a beneficial effect, and the inflammatory parameters showed a marked decrease within 12 days.

One patient in the low-dose group died at day 4 due to fulminant pulmonary embolism—presumably because of disseminated intravascular coagulation, since the repetitive clotting of the hemofiltration lines was observed previous to the patient´s death. The mortality (one in eight) was lower than was expected and calculated by the SOFA score. Other sources report higher mortality rates: (i) the ICNARC (intensive care national audit and research center) covers 5578 patients from the United Kingdom and reported a mortality rate of 67.4% in 1795 patients receiving advanced respiratory support, and a mortality rate of 80.1% in 558 patients receiving any renal support [19]. (ii) An early Chinese study reported a mortality rate of 97% (31 out of 32 patients) in those with invasive mechanical ventilation, based on a total of 191 patients [20]. (iii) Another Chinese study involved 710 COVID-19 patients, of whom 52 were admitted to an ICU and 22 eventually required mechanical ventilation. Of these, 19 (86%) died [21]. (iv) The very first data from the US-24 patients admitted to the ICU at nine Seattle-area hospitals, of whom 18 required mechanical ventilation, showed a mortality rate of 50% (12 out of 24) [22]. (v) The most recent data from the New York City area, including 5700 patients, showed a mortality of 88% in those who were mechanically ventilated [23]. At the ICU of the University Medical Center Hamburg-Eppendorf, 53 patients fulfilled similar criteria as in this case series—a diagnosis of COVID-19, mechanical ventilation, and hypotension with vasopressors required to maintain a mean arterial pressure of ≥65 mmHg. A total of 22 out of these 53 patients died during the hospital stay, corresponding to a mortality rate of 41.5%. Regarding only those patients with acute renal failure in need of dialysis (as five out of eight patients in this case series), the rate increased to 60.6%. Of interest, a steep increase in the DPP3 levels in deceased patient 4 was observed at day 3. DPP-3 was introduced as an independent prognostic marker which is not altered or influenced by Adrecizumab. Just recently, Mebazaa and coworkers have associated high DPP3 levels in critically ill patients with sepsis and cardiogenic shock with reduced cardiac output and low left ventricular function, a high SOFA and liver SOFA score, and short-term mortality [12,13,14,15]. The authors assume that DPP3-mediated impaired prognosis is an independent disease mechanism which cannot be targeted with Adrecizumab or other supportive therapies.

The dosing of the antibody was applied according to the absolute value and the dynamics of bio-ADM in longitudinal measurements. It remains unclear whether the outcomes in the two patients of the low-dose (4 mg/kg) group (including the deceased patient) would differ if they had received the regular dose. It also remains unclear whether the high prevalence of pre-existing RAS-inhibiting therapy (in seven out of eight patients) is an incidental finding.

### 4.2. Other Case Series in Critically Ill COVID-19 Patients

Just weeks ago, a Chinese group reported a case series using convalescent plasma in five critically ill patients with COVID-19 [1]. While their findings were promising, and we follow a similar line of analysis, a direct comparison between both case series falls short. Disease severity in this report was much more pronounced; all eight patients had extensive pre-existing conditions (see Table 1), while within the other report four out of five patients had no co-existing disease at all. This is also mirrored in the median baseline SOFA score, which was 3 in the Chinese report but 12.5 in our report. Moreover, PaO2/FiO2 was lower in our patients, and all our patients were in shock and had vasopressor demand. In addition, six of our patients had renal failure (five with renal replacement therapy) and one patient had liver failure, while these conditions were not seen prior to experimental therapy in the other report.

### 4.3. Adrecizumab and Shock

**Figure 3:**
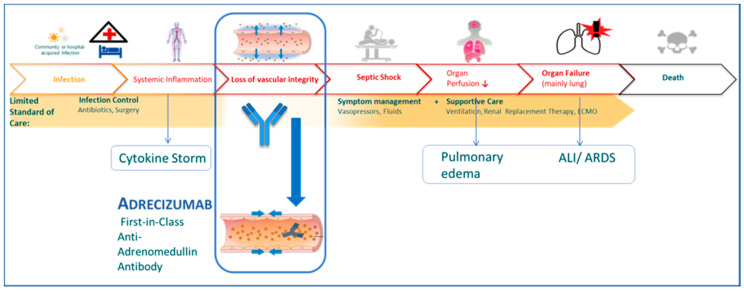
Hypothesis of the loss of vascular integrity preceding septic shock, and Adrecizumab improving endothelial function and vascular integrity in critically ill patients with COVID-19. COVID-19 = coronavirus disease 2019; ALI = acute lung injury; ARDS = acute respiratory distress syndrome.

The loss of vascular integrity plays a pivotal role in the development of vascular leakage and organ dysfunction leading to septic shock [24,25,26]. Several animal studies have proven that ADM shows strong anti-inflammatory properties, improves the cardiomyocyte survival in myocardial ischemia, and has a marked anti-apoptotic effect on cardiomyocytes [27,28]. Moreover, it was demonstrated that ADM infusion attenuates ventilator-induced lung injury by reducing lung hyperpermeability, leucocyte recruitment to the alveolar space, and the deterioration of the systemic microcirculation [29]. While it was recognized that the therapeutic administration of adrenomedullin is not feasible, Adrecizumab acts as a long-lasting plasma ADM enhancer [7,30,31,32,33,34]. In preclinical studies, Adrecizumab reduced the mortality from sepsis and positively impacted the vasoactive adrenomedullin system, leading to the stabilization of blood pressure and renal function and improved catecholamine responsiveness, while the results of the phase 2 trial are still to be reported [7,9,24,25]. As idealized in Figure 3, we hypothesize that the loss of vascular integrity precedes septic shock in COVID-19, and that Adrecizumab is capable to improve endothelial function and vascular integrity in critically ill patients with COVID-19. Importantly, Adrecizumab was claimed to have a long half-life of 14 days, so the therapeutic effect may last up to 8 weeks. Nevertheless, in most of the patients the bio-ADM levels returned to the baseline values within 12 days, which might indicate a shorter duration of effect in septic shock.

### 4.4. Study Limitations

Our report has some limitations that need to be addressed. As a case series, it included no controls, so definite statements regarding the efficacy of Adrecizumab need to be demonstrated in a randomized trial. Furthermore, it remains unclear if the seven surviving patients would have improved without Adrecizumab, although the rapid improvement in PaO2/FiO2 in six of the eight patients and the rapid improvement in the disease severity score in seven of the eight patients, is an encouraging finding. Finally, the influence of the other experimental therapies—lopinavir/ritonavir and CytoSorb^®^—remain unclear. While two patients received both therapies, including the deceased patient 4, another patient received lopinavir/ritonavir alone.

## 5. Conclusions

In this preliminary uncontrolled case series of eight extreme-critically ill patients with COVID-19 and ARDS, the administration of the non-neutralizing anti-ADM antibody Adrecizumab was followed by a favorable outcome. Although the non-controlled design and the small sample size preclude any definitive statement about the potential efficacy of Adrecizumab in critically ill COVID-19 patients, the result of this case series is encouraging.

## Figures and Tables

**Table 1:** Clinical characteristics of the 8 treated patients.

Characteristic	Patient 1	Patient 2	Patient 3	Patient 4	Patient 5	Patient 6	Patient 7	Patient 8
Sex	Male	Male	Male	Male	Male	Male	Male	Female
Age	54	61	73	71	31	76	58	68
BMI	29.2	31.9	26.4	32.7	41.4	30.9	27.7	39.1
SAPS II score on admission to ICU	32	55	37	43	20	64	40	35
Chronic diseases	T2DM Hypertension Previous stroke	T2DM Hypertension Sarcoidosis	T2DM Hypertension	Hypertension Atrial fibrillation Tuberculosis with lung resection	T2DM	T2DM Hypertension Atrial fibrillation Previous stroke Hemochromatosis Rheumatoid arthritis Sleep apnea	Hypertension Atrial Fibrillation	T2DM Hypertension Hypothyroidism Renal Failure Myasthenia gravis Neuromyelitis optica
Pre-existing RAS-I medication	ACE-inhibitor	ACE-inhibitor	ACE-inhibitor	Angiotensin receptor blocker	None	Angiotensin receptor blocker	ACE-inhibitor	ACE-inhibitor
Complication prior to Adrecizumab therapy	Bacterial pneumonia Severe ARDS Shock	Bacterial pneumonia Severe ARDS Renal failure with need for renal replacement therapy Shock	Severe ARDS Shock	Bacterial pneumonia Severe ARDS Renal failure with need for renal replacement therapy Liver failure Shock	Severe ARDS Renal failure with need of renal replacement therapy Shock	Bacterial pneumonia Severe ARDS Renal failure with need for renal replacement therapy Shock	Bacterial pneumonia Severe ARDS Renal failure Shock	Bacterial pneumonia Severe ARDS Renal failure with need of renal replacement therapy Shock
Days between symptoms onset and positive testing	2	5	14	4	4	2	1	
Days between symptom onset and admission	9	5	15	3	5	3	15	3
Days between ICU admission and Adrecizumab therapy	1	2	2	2	1	1	1	3
Most severe disease classification	Critical	Critical	Critical	Critical	Critical	Critical	Critical	Critical
Prior CPR	No	No	No	No	No	No	Yes	No
Prior treatment with antivirals	Lopinavir/ritonavir	Lopinavir/ritonavir	None	Lopinavir/ritonavir	None	None	None	None
Other experimental therapies	None	CytoSorb^®^	None	CytoSorb^®^	None	None	None	None

BMI = body-mass-index; T2DM = type 2 diabetes mellitus; RAS = renin-angiotensin-system; ICU = intensive care unit; CPR = cardiopulmonary resuscitation.

**Table 2:** Dose of Adrecizumab administered and course of therapy-guiding biomarkers bio-ADM and DPP-3.

Characteristic	Patient 1	Patient 2	Patient 3	Patient 4	Patient 5	Patient 6	Patient 7	Patient 8
Body weight [kg]	100	100	75	100	125	98	100	100
Dosing group	high-dose [8 mg/kg bw]	high-dose [8 mg/kg bw]	high-dose [8 mg/kg bw]	low-dose [4 mg/kg bw]	high-dose [8 mg/kg bw]	low-dose [4 mg/kg bw]	high-dose [8 mg/kg bw]	high-dose [8 mg/kg bw]
Dose Adrecizumab [mg]	800	800	600	400	1000	400	800	800
Bio-ADM [pg/mL] before Adrecizumab	63.9	64.9	53.9	53.0	102.6	191.3	90.7	170.3
day 1	264.5	377.2	403.9	239.4	226.1	678.9	388.8	515.0
day 2	244.6	n/a	281.5	312.3	271.1	383.8	257.6	n/a
day 3	235.6	274.7	200.2	171.8	239.8	209.9	231.1	224.8
day 5	123.5	194.9	157.7	n/a	246.1	130.3	167.9	n/a
day 7	63.9	113.8	143.0	n/a	155.0	118.9	n/a	n/a
day 10–2	42.3	130.6	53.8	n/a	134.0	170.3	92.0	n/a
DPP-3 [ng/mL] before Adrecizumab	6.92	19.3	17.9	19.0	19.0	23.5	18.3	12.5
day 1	7.38	18.9	12.8	17.7	n/a	n/a	n/a	n/a
day 3	13.22	7.84	n/a	135.7	n/a	n/a	n/a	n/a
day 5–10	9.90	n/a	n/a	>150	n/a	23.5	n/a	n/a

Bio-ADM = bioactive adrenomedullin; DPP-3 = dipeptidyl peptidase-3; kg = kilogram; bw = body weight.

**Table 3:** Comparison of clinical parameters and score before and after the Adrecizumab administration.

Characteristic	Patient 1	Patient 2	Patient 3	Patient 4	Patient 5	Patient 6	Patient 7	Patient 8
Days of follow-up	29	27	24	4	21	21	20	13
Current status as of 15 April 2020	Transferred to normal ward	Alive; still receiving mechanical ventilation	Transferred to normal ward	Deceased–81 h after intervention	Alive; still receiving mechanical ventilation	Alive; still receiving mechanical ventilation	Transferred to normal ward	Alive; still receiving mechanical ventilation
Mechanical ventilation								
Onset days before Adrecizumab	1	2	1	2	1	1	1	1
Status	Extubated	De-escalation from BIPAP to intermittent CPAP	Extubated	Deceased	De-escalation from BIPAP to intermittent CPAP	De-escalation from BIPAP to intermittent CPAP	Extubated	De-escalation from BIPAP to intermittent CPAP
ECMO								
Onset before/after Adrecizumab	Not received	Same day	Not received	Not received	Same day	Not received	Same day	Not received
Removal, days after Adrecizumab	n/a	Still in use	n/a	n/a	19	n/a	6	n/a
PaO_2_/FiO_2_								
before Adrecizumab	126	90	181	108	107	213	75	160
best value within 12 h	244	127	224	122	133	215	113	241
mean value day 1	204	105	184	142	81	171	132	168
mean value day 2	186	96	161	116	87	162	163	166
mean value day 3	218	106	177	93	78	165	155	185
mean value day 5	216	122	190	n/a	171	181	192	196
mean value day 7	225	98	209	n/a	162	97	316	220
mean value day 10	259	78	226	n/a	168	134	348	236
mean value day 12	249	84	240	n/a	176	157	295	230
Noradrenaline [µg/kg/min]								
before Adrecizumab	0.02	0.20	0.14	0.14	0.11	0.16	0.52	0.06
highest dose day 1	0.10	0.96	0.17	0.40	0.11	0.30	0.33	0.07
highest dose day 2	0.14	0.48	0.16	1.60	0.11	0.11	0.28	0.04
highest dose day 3	0.12	0.48	0.15	4.00	0.13	0.07	0.09	0.06
highest dose day 5	0	0.44	0.26	n/a	0.10	0.09	0.10	0.08
highest dose day 7	0	0.20	0.16	n/a	0.09	0.20	0.11	0.02
highest dose day 10	0.25	0.44	0	n/a	0.23	0.31	0	0
highest dose day 12	0	0.40	0	n/a	0.25	0.37	0	0
SOFA score								
before Adrecizumab	12	14	12	12	16	12	13	14
day 1	11	16	12	16	16	12	12	14
day 2	10	17	11	18	16	12	14	13
day 3	10	17	11	n/a	15	11	14	13
day 5	10	18	10	n/a	14	13	11	12
day 7	6	17	10	n/a	14	12	13	13
day 10	7	19	6	n/a	12	13	10	4
day 12	9	18	6	n/a	14	13	5	7
C-reactive protein [mg/L]; Reference value < 5 mg/L								
before Adrecizumab	105	186	86	304	72	284	137	37
day 1	151	277	186	339	80	213	160	24
day 2	203	286	279	334	42	239	136	47
day 3	210	189	307	266	27	224	122	43
day 5	82	61	296	n/a	45	100	91	89
day 7	61	108	261	n/a	20	292	91	205
day 10	27	213	97	n/a	22	286	56	150
day 12	29	141	51	n/a	14	122	35	72
Procalcitonin [µg/L]; Reference value < 0.5µg/L								
before Adrecizumab	0.47	2.56	n/a	1297	2.29	n/a	2.76	0.15
day 1	0.55	17.33	0.49	n/a	n/a	4.08	2.37	0.25
day 2	0.49	14.8	n/a	456.8	1.51	9.86	1.64	0.32
day 3	0.6	9.33	0.48	413.1	n/a	7.74	1.38	0.22
day 5	0.35	4.96	n/a	n/a	0.79	4.02	0.77	0.25
day 7	0.23	2.27	n/a	n/a	0.51	3.08	0.51	0.39
day 10	0.1	1.87	0.23	n/a	0.67	4.21	0.26	0.36
day 12	0.12	1.75	0.20	n/a	0.86	3.35	0.20	0.20
Interleukin-6 [ng/L]; Reference value < 7 ng/L								
before Adrecizumab	129	18,825	n/a	1297	304	n/a	364	33
day 1	426	781	1052	n/a	50	n/a	427	93
day 2	132	106	n/a	457	46	n/a	232	79
day 3	179	37	421	413	41	n/a	173	101
day 5	62	104	382	n/a	43	n/a	131	41
day 7	62	132	192	n/a	15	1078	84	67
day 10	33	129	34	n/a	36	335	32	35
day 12	24	94	63	n/a	49	58	21	15
Lactate [mmol/L]; Reference value 0.5–2.2 mmol/L								
before Adrecizumab	1.1	3.4	0.9	1.3	2.3	3.0	2.2	0.7
day 1	1.4	3.6	1.0	2.5	2.4	1.9	1.5	0.7
day 2	1.2	3.0	1.4	7.0	1.8	2.1	1.0	0.6
day 3	1.3	2.2	1.5	12.0	2.2	1.3	0.9	1.0
day 5	1.4	1.7	1.3	n/a	1.5	2.0	1.0	0.8
day 7	0.9	1.5	1.3	n/a	1.2	1.3	1.0	0.8
day 10	n/a	1.8	1.2	n/a	1.6	1.6	1.0	0.4
day 12	n/a	1.8	1.1	n/a	1.4	1.1	1.2	0.5

ECMO = extracorporeal membrane oxygenation; SOFA = sequential organ failure assessment; kg = kilogram; h = hours.

**Table 4:** Comparison of the disease severity score before and after the Adrecizumab administration.

	Patient 1	Patient 2	Patient 3	Patient 4	Patient 5	Patient 6	Patient 7	Patient 8
Vital and hospitalization status								
Before Adrecizumab	3	3	3	3	3	3	3	3
day 1	3	3	3	3	3	3	3	3
day 3	3	3	3	3	3	3	3	3
day 7	3	3	3	5	3	3	3	3
day 12	3	3	3	n/a	3	3	3	3
last day of follow-up	1	3	1	n/a	3	3	1	3
Circulation status								
Before Adrecizumab	1	2	2	2	2	2	3	1
day 1	1	3	2	2	2	2	2	1
day 3	2	3	2	3	2	1	1	1
day 7	0	2	2	3	1	2	2	1
day 12	0	2	0	n/a	2	2	0	0
last day of follow-up	0	3	0	n/a	0	3	0	0
Ventilation status								
Before Adrecizumab	3	3	3	3	4	3	4	2
day 1	3	4	2	3	4	3	4	2
day 3	3	4	3	3	4	3	4	2
day 7	3	4	3	3	4	3	2	2
day 12	1	4	0	n/a	4	3	0	2
last day of follow-up	0	4	0	n/a	2	2	0	2
Mean PaO_2_/FiO_2_								
Before Adrecizumab	2	3	2	2	2	1	3	2
day 1	1	2	2	2	3	2	2	2
day 3	1	2	2	3	3	2	2	2
day 7	1	3	1	3	2	3	0	1
day 12	1	3	1	n/a	2	2	1	1
last day of follow-up	0	3	0	n/a	1	2	0	0
Total score								
Before Adrecizumab	9	11	10	10	11	9	13	8
day 1	8	12	9	10	12	10	11	8
day 3	9	12	10	12	12	9	10	8
day 7	7	12	9	14	10	11	7	7
day 12	5	12	4	n/a	11	10	4	6
last day of follow-up	1	13	1	n/a	6	10	1	5

Scoring system: maximum 16, minimum 0, with higher scores indicating more severe illness. Vital and hospitalization status: deceased (5), on ICU ward (3), on normal ward (1), discharged (0). Circulation: VA-ECMO (4), max. noradrenaline > 0.40 µg/kg/min (3), max. noradrenaline 0.40–0.10 µg/kg/min (2), max. noradrenaline < 0.10 µg/kg/min (1), no vasopressor (0). Ventilation status: VV-ECMO (4), mechanical ventilation BIPAP (3), intermittent mechanical ventilation CPAP (2), non-invasive ventilation (1), no non-invasive ventilation (0). Mean PaO2/FiO2: ≤ 100 (3), 101–200 (2), 201–300 (1), ≥300 (0).
